# Registered nurse–patient communication and decision-making in primary care consultations: a scoping review

**DOI:** 10.1186/s12912-026-04937-w

**Published:** 2026-06-23

**Authors:** Sofia Östensson, Annelie J. Sundler, Karin Bergman, Laura Darcy, Lotta Saarnio Huttu, Lena Hedén, Sandra Van Dulmen, Inger K. Holmström, Malin Östman

**Affiliations:** 1 Faculty of Caring Science, Work Life and Social Welfare, University of Borås, Borås, Sweden; 2 The Swedish Red Cross University, Stockholm, Sweden; 3 Nivel (Netherlands Institute for Health Services Research), Utrecht, The Netherlands; 4 Department of Primary and Community Care, Radboud Institute for Health Sciences, Radboud University Medical Center, Nijmegen, The Netherlands; 5 Department of Health, Innovation and Design, Mälardalen University, Västerås, Sweden; 6 Department of Public Health and Caring Sciences, Uppsala University, Uppsala, Sweden; 7 Research, Education, Development & Innovation, Primary Health Care, Region Västra Götaland, Gothenburg, Sweden; 8 General Practice/Family Medicine, School of Public Health and Community Medicine, Institute of Medicine, Sahlgrenska Academy, University of Gothenburg, Gothenburg, Sweden

**Keywords:** Communication, Consultation, Decision-making, Interaction, Primary care nursing, Primary health care, Registered nurse, Scoping review

## Abstract

**Background:**

Internationally, interest in the expanding role of registered nurse–led consultations in primary care is increasing. This role requires effective communication to support triage and clinical decision-making. While physician‒patient communication has been extensively studied, research on registered nurse‒patient communication remains limited, particularly in primary care. Therefore, the aim of this scoping review was to map and describe how registered nurse–patient communication and decision-making in primary care consultations are characterised in the existing literature, based on observations of clinical practice.

**Methods:**

This scoping review included a systematic literature search conducted in March 2025 in the PubMed, CINAHL, Web of Science and Scopus databases. A total of 12,066 records were identified and screened. The review followed the methodological framework of Arksey and O’Malley and adhered to the PRISMA-ScR reporting guidelines.

**Results:**

A total of 16 studies were included. Registered nurse‒patient communication was observed using audio- and video-recorded observations and participant observations during telephone consultations (*n* = 5), chronic disease management consultations (*n* = 3), mental health consultations (*n* = 1) and walk-in clinic consultations (*n* = 7). Findings on communication and decision-making were organised into four themes related to the key characteristics of exploring patients’ concerns, observing and assessing patients’ health status, tailoring communication, and facilitating decision-making with patients.

**Conclusion:**

The findings indicate that registered nurses use various communication skills during different phases of consultations. However, comprehensive studies on registered nurse–patient communication and decision-making in primary care are lacking. This highlights the need for future in-depth and comprehensive studies on registered nurse–patient communication in primary care to inform both clinical practices and nursing education.

**Trial and protocol registration:**

Prospero registration: PROSPERO 06 June 2023 CRD42023425582. Available from:https://www.crd.york.ac.uk/prospero/display_record.php?ID=CRD42023425582.

**Supplementary Information:**

The online version contains supplementary material available at 10.1186/s12912-026-04937-w.

## Background

Primary care is the first point of contact in the healthcare system and is essential for managing acute, chronic, and preventive health needs. The roles and responsibilities of registered nurses (RNs) in primary care vary internationally, ranging from performing specific tasks and providing general support for general practitioners (GPs) [1, 2] to RN–led consultations to complement or replace GPs and taking on roles traditionally performed by GPs [3, 4]. RN–led consultations in patient management and triage require advanced competencies in communication and clinical decision-making.

Appropriate patient management in RN–led consultations is highly dependent on the quality of communication with patients. For at least two decades, communication skills training has been recognized internationally as a critical component of medical education and GP training [5]. Comprehensive models for structuring medical interviews and building relationships, such as the Calgary–Cambridge guide, have been developed on the basis of empirical evidence. However, this emphasis is not as prominent in nursing practices and education. As RN–led consultations have the potential to increase the efficiency of patient management [4], there is a need to summarise existing research on key aspects of RN–led communication and decision-making in primary care consultations.

Communication and interaction in RN–patient encounters are well-known key components of effective nursing care in general [6]. RN–patient communication is multifaceted and involves more than just an exchange of information. An RN’s communication with the patient is the basis for an exploration and understanding of the patient’s health concerns [7]. It can lead to better health outcomes, more effective processing of information, improved adherence to treatment and advice, and greater patient satisfaction [8, 9].

RN–led consultations must ensure efficient patient management and triage, require communication for information exchange and make decisions on the basis of the urgency and severity of the patient’s health condition. In primary care, RNs generally play a crucial role in the initial assessment, either by a telephone nurse [10] or face-to-face in primary care consultations with an RN or a GP. Healthcare professionals must be able to identify patients’ often complex needs and preference-sensitive care demands and to make priorities accordingly [7]. An early observational study on clinical communication, published outside the inclusion period of this review, described cognitive processes involved in clinical decision-making. The authors reported that RNs used multiple approaches when explaining how they reached clinical decisions [11]. A more recent study by Martinez-Angelo et al. [12] illustrates the complexity and power dynamics of RN–patient communication in primary care, as reported in interviews. This complexity may be influenced by how health professional develop and apply communication skills and shared decision-making competencies in daily primary care practice [13]. While communication in physician–patient consultations is well known as fundamental for information exchange, understanding needs, patient involvement, and empowerment [8, 9, 14], insights from observational studies further exploring the dynamics and challenges related to RN–patient communication and decision-making in primary care consultations remains limited [6]. Hither to, no previous review has focused on observational studies on RN–patient communication and decision-making in primary care practices. To address this issue and guide future communication research in the context of primary care nursing practices, this review was conducted. Therefore, the aim of this scoping review was to map and describe how registered nurse–patient communication and decision-making in primary care consultations are characterised in the existing literature, based on observations of clinical practice.

## Methods

### Design

A scoping review with a systematic literature search was conducted following the methodology described by Arksey and O’Malley [15]. Scoping reviews aim to map concepts and evidence in a specific field and can include broader questions than systematic reviews do. The review followed the five steps described by the Joanna Briggs Institute and Arksey and O’Malley [15, 16]. This review was based exclusively on empirical research papers and used both quantitative and qualitative designs. The steps are as follows: (1) identifying the research question, (2) identifying relevant studies, (3) study selection, (4) charting the data, and (5) collating, summarising and reporting the results. To establish clear and meaningful aim and eligibility criteria, the population/concept/context (PCC) framework was used [17]. Owing to the great variability in nursing roles and responsibilities in primary care [18], the term RN (population) was used for all types of registered nurses, including practice nurses and nurse practitioners working in primary care (context), with a specific focus on observed communication and decision-making (concepts). The review was registered in Prospero prior to the start of the study. The description of the study has been further refined during the process.

### Search methods

A systematic search strategy was conducted to identify relevant studies with the help of a professional librarian. The search was carried out in PubMed, CINAHL, Web of Science and Scopus and manually via reference lists. First, a broad literature search was conducted using search terms based on MeSH terms, including ‘Primary healthcare’, ‘Nurses’, ‘Decision-making’, ‘Clinical reasoning’, ‘Health communication’ and ‘Nurse–patient consultations’; see Supplementary file 1.

### Inclusion and exclusion criteria

To ensure the inclusion of empirical studies on observed RN–patient communication and decision-making, the inclusion criteria were relevant to the study’s aim: peer-reviewed empirical studies written in English. The initial search included peer-reviewed studies published between January 1998 and March 2025. This broad time frame was intentionally chosen to ensure comprehensive coverage and to avoid unintentional exclusion of relevant literature, as no prior review studies were identified in the field. During the process, a priori decision was made to limit inclusion to studies published from 2005 onwards. The final inclusion period therefore comprised studies published between 2005 and March 2025. This refinement did not affect the scope of the review but enhanced the relevance and analytical coherence of the included studies. We excluded review studies, single case studies, theoretical studies, unpublished materials, “grey” literature, dissertations, conference presentations, and empirical studies if the results were not specific to RNs.

### Search outcome

**Fig. 1:**
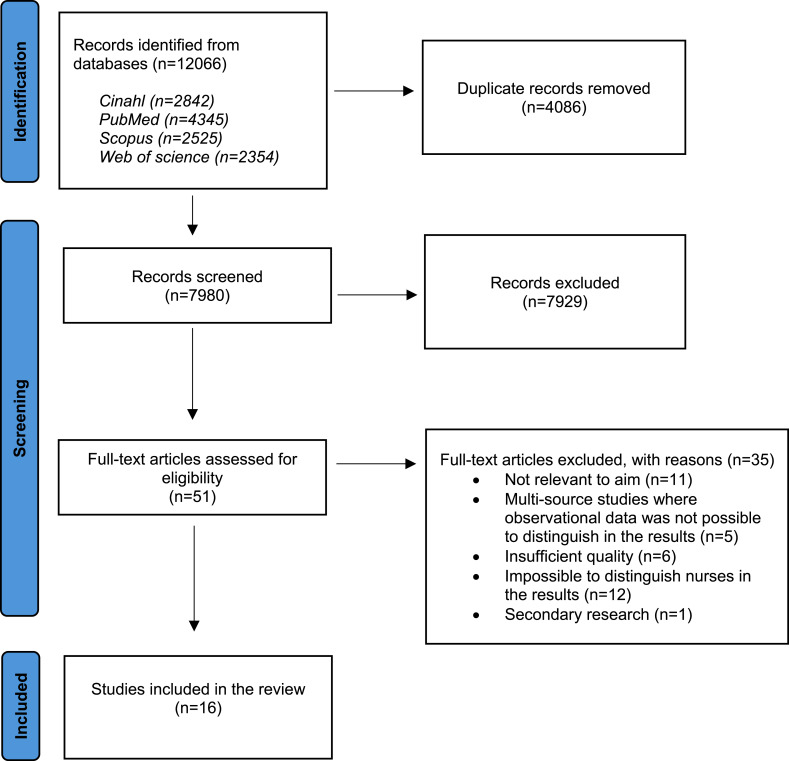
Flow diagram of the literature search

A total of 12,066 records were identified. The records were screened for duplicates. The screening process was subsequently conducted with the Rayyan web application [19]. First, the records were screened by title and then by abstract. In the initial screening, four of the authors screened 100 records together, and subsequent joint discussions were held to address uncertainties and ensure adherence to the inclusion and exclusion criteria. Thereafter, three of the authors independently screened 1000 records to establish agreement on the screening. Discussions took place to resolve uncertainties and to ensure agreement. Two authors then independently screened the remaining records for reliability. If the relevance of a study was unclear from the abstract, then the full article was read. A total of 51 studies were read in full, 35 of which were excluded. For example, studies involving a mixed group of professionals, both RNs and other healthcare professionals, were excluded when the results specific to RNs could not be separately identified. The Preferred Reporting Items for Systematic Reviews and Meta-Analyses (PRISMA-ScR) checklist [20] was used, and the literature search and screening were documented as a Prisma flow diagram (see Fig. 1).

### Data charting and collation of results

Data were charted from the included studies using a predefined charting form. Both study characteristics and study findings relevant to the review questions were extracted. Charted study characteristics were summarised in a table describing study aim; methods, study design and data sources; setting and sample. An overview of the included studies is presented in Table 1.

**Table 1:** Overview of study characteristics

Study	Country	Aim	Methods/study design and data sources	Setting and sample
Barrat J. & Thomas N. (2019) Nurse practitioner consultations in primary health care: an observational interaction analysis of social interactions and consultation outcomes.	UK	To determine the discrete nature of social interactions occurring in nurse practitioner consultations, and to investigate the relationship between consultation social interaction styles (biomedical and patient-centred) and the outcomes of patient satisfaction, patient enablement and consultation time lengths.	Quantitative study Data gathered from video-recorded observations (*n* = 30) and questionnaires (*n* = 26)	Primary care, NP consultations *Participants*: 3 NPs (no information about gender), 30 patients (adults *n* = 20/children *n* = 10, 24 female/6 male)
Barrat J. (2005) A case study of styles of patient self-presentation in the nurse practitioner primary health care consultation.	UK	To identify whether there were any different styles of patient self-presentation in the nurse practitioner consultation, and to describe their effects, if any, on the process and outcomes of the nurse practitioner consultation.	Qualitative study Observation, interviews, field journal Data gathered from observations (*n* = 15) and field journals (*n* = 15)	Primary care and nurse–led primary healthcare walk-in clinic. NP consultations *Participants*: 6 NPs (female), 15 patients (9 female/6 male)
Collins S. (2005) Explanations in consultations: the combined effectiveness of doctors’ and nurses’ communication with patients.	UK	To consider how explanations are deployed in patients’ communication with doctors and nurses.	Qualitative study Data gathered from audio-recorded consultations (*n* = 38)	Primary care Diabetes management *Participants*: 5 nurses (female), 6 physicians (1 female/5 male) and 23 patients (no information about gender)
de Almeida P.A. (2025) Mental health nursing consultations in Brazilian primary care: analysis of proposed competencies for advanced practice	Brazil	To analysed nursing consultations in mental health within PHC and investigated whether nurses possess the care management skills pro- posed for the Advanced Nursing Practices.	Quantitative and qualitative study. Data gathered video recorded observations (*n* = 49)	Primary care, ANP mental health consultations. *Participants*: 21 ANPs (female), 49 patients
James S. et al. (2020) Nonverbal communication between registered nurses and patients during chronic disease management consultations: Observations from general practice.	Australia	This study explores nonverbal communication behaviours between general practice nurses and patients during chronic disease consultations.	Quantitative study Data gathered from video recordings (*n* = 36)	Primary healthcare, chronic disease management *Participants*: 14 general practice nurses (female) and 36 patients (20 female/16 male)
Leppänen V. (2010) Power in telephone-advice nursing.	Sweden	To develop a framework for analysing how power operates in nurse–patient interaction and to empirically analyse power in one specific context of telephone-advice nursing in Swedish primary care.	Qualitative study Data gathered from audio recordings (*n* = 276)	Primary care, telephone-advice nursing *Participants*: 13 nurses (audio recorded) and 18 nurses (interviews) (*n* = 31) (no information about gender)
Macdonald L. et al. (2013) Nurse-patient communication in primary care diabetes management: an exploratory study.	New Zealand	To examine the actual talk and perspectives of nurses and patients who were newly diagnosed with diabetes in order to describe the features of effective interaction and to identify areas for reflection and possible improvements to practice.	Qualitative study Data gathered from video and audio recordings (*n* = 35)	Primary care Diabetes management *Participants*: 10 nurses (no information about gender) and 18 patients (10 female, 8 male)
McCaughan D. et al. (2005) Nurse practitioner and practice nurses’ use of research information in clinical decision-making findings from an exploratory study.	UK	To describe the decisions made by nurses working in general practice and the sources of information they use to underpin those decisions.	Qualitative study Data gathered from observation, interviews, documentary/resource audit	Primary care, routine work *Participants*: 29 nurses and 4 NPs (no information about gender)
Murdoch J. et al. (2015) The impact of using computer decision-support software in primary care nurse-led telephone triage: interactional dilemmas and conversational.	UK	To compare doctors’ and nurses’ communication with patients in primary care telephone triage consultations.	Qualitative study Data gathered from audio recorded calls (*n* = 22), video recordings (*n* = 10)	Primary care, telephone triage *Participants*: 4 nurses and 22 patients (no information about gender)
Murdoch J. et al. (2014). Question design in nurse-led and GP-led telephone triage for same-day appointment requests: a comparative investigation	UK	To compare doctors’ and nurses’ communication with patients in primary care telephone triage consultations.	Quantitative study Data gathered from audio recordings (*n* = 51), including video recordings (*n* = 10)	Primary care telephone triage *Participants*: 22 nurses (17 female/5 male), 29 GPs (23 female, 6 male) and 51 patients (no information about gender)
Noordman J. et al. (2014) Effects of video-feedback on the communication, clinical competence and motivational interviewing skills of practice nurses: a pre-test post test control group study.	Netherlands	To examine the effects of individual video feedback on generic communication skills, clinical competence (i.e. adherence to practice guidelines) and motivational interviewing skills of experienced practice nurses working in primary care.	Quantitative non-randomized study Data gathered from video recording (*n* = 325), pre- and post-test	Primary care, practice nurses’ consultations *Participants*: 17 nurses (female) and 163 patients (both female and male)
Paniagua H. (2011) Advanced nurse practitioners and GPs: What is the difference?	UK	To determine the role of the ANP.	Qualitative study Data gathered from video recorded consultations (*n* = 37)	Primary care evening surgeries, ANP consultations *Participants*: 24 ANPs and 13 GPs (no information about gender)
Reblora J.M. et al. (2020) “The same but different” Triaging in primary healthcare settings: A focused ethnography study.	Singapore	To explore the experiences of nurses working at triage stations at primary healthcare centres.	Qualitative study Data gathered from observations (*n* = 70 h) In-depth interviews (*n* = 22)	Primary care, RN triage *Participants*: 22 RNs (female)
Seale C. et al. (2005) Comparison of GP and nurse practitioner consultations: an observational study.	UK	To discover what nurse practitioners, do with the extra time, and how their consultations differ from those of GPs.	Quantitative descriptive study Data gathered from audio-recorded consultations (*n* = 55) Matched consultations (*n* = 18)	Primary care, NP consultation *Participants*: 33 NPs and 22 GPs general practitioners. Matched consultations: 9 nurses (female) and 8 GPs (4 female/4 male)
Spek et al. 2023 Displaying concerns within telephone triage conversations of callers with chest discomfort in out-of-hours primary care: A conversation analytic study	Netherlands	To better understand the interactional implications of discussing concerns during triage conversations between people who called the OHS-PC for chest discomfort and triage nurses who use the NTS tool.	Quantitative study Data gathered from audio-recorded consultations (*n* = 68), selected consultation with concerns (*n* = 35)	Primary care telephone triage. RN *Participants*: No information of RN
Vilstrup E. et al. (2019) Communicative characteristics of general practitioner-led and nurse-led telephone triage at two Danish out-of-hours services: an observational study of 200 recorded calls.	Denmark	To compare communicative parameters in GP-led and nurse-led OOH telephone triage, and to discuss differences in relation to patient-centred communication and safety issues.	Quantitative descriptive study Data gathered from 200 audio-recorded telephone triage conversations with GPs (*n* = 100) and nurses (*n* = 100)	Primary care, telephone triage by GPs or nurses *Participants*: 100 nurses and 100 GPs (no information about gender)

Abbreviations: ANP= Advanced Nurse Practitioner; GP= General Practitioner; NP= Nurse Practitioner; RN= Registered Nurse

In line with the scoping review approach described by Arksey and O’Malley [15], findings from the included studies were charted and mapped into thematic areas related to RN–patient communication and decision-making. Three of the authors (SÖ, MÖ, AS) read all included articles and extracted relevant findings guided by the review questions. The charted data were summarised and organized into thematic areas by identifying patterns across studies. This analytic process involved mapping findings into themes reflecting how RN–patient communication and decision-making were described in the literature. The themes were discussed among the authors with a focus on identifying key characteristics of RN–patient communication and decision-making. To support the collation and presentation of results, a patterning chart of key themes was developed, see Table 2, illustrating the distribution of themes across the included articles [21].

**Table 2:** Key themes patterning chart (*n* = 16)

Articles	Exploring patients’ concerns	Observing and assessing patient’s health status	Tailoring the communi- cation	Facilitating decision-making with patients
Barrat J. & Thomas N. (2019) Nurse practitioner consultations in primary health care: an observational interaction analysis of social interactions and consultation outcomes.	x		x	x
Barratt J. (2005) A case study of styles of patient self-presentation in the nurse practitioner primary health care consultation.	x	x	x	x
Collins S. (2005) Explanations in consultations: the combined effectiveness of doctors’ and nurses’ communication with patients.	x		x	x
de Almeida P.A. et al. (2025) Mental health nursing consultations in Brazilian primary care: analysis of proposed	x		x	x
James S. et al. (2020) Nonverbal communication between registered nurses and patients during chronic disease management consultations: Observations		x		x
Leppänen V. (2010) Power in telephone-advice nursing.	x			x
Macdonald L. et al. (2013) Nurse-patient communication in primary care diabetes management: an exploratory study.	x	x		x
McCaughan D. et al. (2005) Nurse practitioner and practice nurses’ use of research information in clinical decision-making findings from an exploratory study.				x
Murdoch J. et al. (2015) The impact of using computer decision-support software in primary care nurse-led telephone triage: interactional dilemmas and conversational.	x			x
Murdoch J. et al. (2014) Question design in nurse-led and GP-led telephone triage for same-day appointment requests: a comparative investigation	x			x
Noordman J. et al. (2014) Effects of video-feedback on the communication, clinical competence and motivational interviewing skills of practice nurses: a pre-test post-test control group study.			x	
Paniagua H. (2011) Advanced nurse practitioners and GPs: What is the difference?	x	x		
Reblora J.M. et al. (2021) “The same but different” Triaging in primary healthcare settings: A focused ethnography study.	x		x	x
Seale C. et al. (2005) Comparison of GP and nurse practitioner consultations: an observational study.	x	x	x	x
Spek M. et al. (2023) Displaying concerns within telephone triage conversations of callers with chest discomfort in out-of-hours primary care: A conversation analytic study	x			x
Vilstrup E. et al. (2019) Communicative characteristics of general practitioner-led and nurse-led telephone triage at two Danish out-of-hours services: an observational study of 200 recorded calls.	x		x	

## Results

### Study characteristics

A total of 16 studies [22–37] were includedpublished between 2005 and 2025 were included. No studies published between 1998 and 2005 met the inclusion criteria.

Study designs included qualitative (*n* = 12), quantitative (*n* = 3) and the combination of qualitative and quantitative approaches (*n* = 1). The data collected in the studies consisted of audio recordings (*n* = 6), video recordings (*n* = 4) participant observations (*n* = 3), and studies combining data from participant observations and video recordings (*n* = 3). The studies originated from the UK (*n* = 8), Netherlands (*n* = 2), New Zealand (*n* = 1), Australia (*n* = 1), Brazil (*n* = 1), Denmark (*n* = 1), Singapore (*n* = 1) and Sweden (*n* = 1). The participating RNs had varying levels of education: registered nurses (*n* = 11), nurse practitioners (*n* = 4), and advanced nurse practitioners (*n* = 2). The RN–patient encounters involved walk-in clinic consultations (*n* = 8), telephone triage consultations (*n* = 5), chronic disease management consultations (*n* = 3), and mental health consultations (*n* = 1).

### Findings on RN–patient communication and decision-making

The charted data on RN–patient communication were summarised into four thematic areas, each reflecting distinct aspects of communication and decision-making practices during consultations. The themes identified were: exploring patients’ concerns (*n* = 13), observing and assessing patients’ health status (*n* = 5), tailoring communication (*n* = 8), and facilitating decision-making with patients (*n* = 13). The distribution of studies across themes illustrates the relative focus of the existing literature. These themes are described in detail below.

### Exploring patients’ concerns

The initial explorations of patients’ concern by RNs is the first thematic area described in a total of thirteen studies [22–25, 27, 28, 30, 31, 33–37]. A wide range of topics and concerns were elicited and addressed during RN consultation, where various communication approaches were employed to facilitate the exploration of patients’ concerns and problems with openness.

Consultations commonly start with the RN inviting patients to provide information about medical conditions, therapeutic regimens or lifestyle [24, 25]. For example, the RN can ask, “How have you been this last year, would you say?” [24]. In the opening phase of the consultation, the RN’s listening was described as crucial. For example, RNs provide space for patients to offer explanations with openness by simply not talking [24, 25]. Understanding the patient’s problem requires the RN to listen and allow the patient to talk freely. The way the patients presented their problems differed; some patients were vague, whereas others were observed to describe their problem in more precise terms and moved towards describing the present conditions more quickly than others did [22, 24, 25, 27, 34]. During this exploration, RNs were found to show their understanding of—or agreement with—the patient’s problem. Without commenting, RNs could use back-channelling, for example, by saying “uhm” to confirm that they were listening [22, 25, 28]. However, active listening was reported to be limited during consultations, with registered nurses paying minimal attention to both verbal and nonverbal communication, as observed in the study by Almeida [25].

The RNs asked questions in different ways. Open-ended questions regarding presenting issues are commonly used to indicate to patients whether they understood or agreed [22, 24, 27]. Closed-ended and leading questions were more common than open-ended questions [22, 35, 37]. Specific questions were used to explore the medical reasons for the visit. By asking one question at a time, the RNs strived to let the patients articulate their problem from their own perspective. If the patients presented their symptoms or problems too briefly, the RNs could repeat their questions in another way or return to the same question at a later point during the consultation. For example, RNs returned to symptoms by summarizing [30, 33] “Okay, so you said it was radiating down your back instead of down your leg, is that right?” [30] In contrast, patient involvement in discussing therapeutic options, planning care, and summarizing the key points of the consultation was infrequent in the study conducted by Almedia et al. [25]. Questions were also asked to obtain confirmation from patients about their problems such as “And you said you had experienced no discharges and no pain [31]? . When focusing on symptoms and problems, RNs could enter into a question-and-answer history format when eliciting further information from patients [23, 27]. Specifically, in telephone triage, such closed-ended questioning was found as a result of the use of a decision support system. When using such a system, RNs must select one of the patient’s problems even if they report several symptoms to conduct their triage. The RN questioning then becomes a result of the system, commonly resulting in a priority of symptom-related questions needing to be asked to identify the problem with the highest level of urgency. The questions were designed to elicit a response that fit the system, and it was not possible to leave the highest-urgency-related questions unanswered. Consequently, RNs’ questions become a result of the system without acknowledging their own competence and communication skills [31, 36]. This may result in patients not receiving adequate responses to their concerns or in RNs focusing solely on the somatic aspects of these concerns. Furthermore, if patients did not receive an appropriate response, they tended to revisit their issues later in the consultation, underscoring a need for a more empathetic and comprehensive response to their concerns. Commonly, RN returned to triage conversations without responding to patients’ concerns [36].

The exploration of patients’ concerns was a result not only of RNs’ communication but also of the communication styles of patients and how they presented their condition [23]. Styles identified were patients seeking treatment, patients being clinical presenters (meaning that they presented their clinical histories), patients wanting to check the severity of their condition and patients who had already anticipated their need for treatment. Patients could also demonstrate flexibility by shifting between different communication styles during consultations. Although RNs predominantly initiated questions, patients could ask questions on their initiative [22, 35, 37]. In contrast, Almedia’s study [25] reported limited efforts by RNs to encourage patients to express their expectations, feelings, and concerns.

The different reported communication styles were related to being either more patient-centred or more biomedical. when exploring patients’ concerns and encourage them to present their problems and associated concerns [22]. In the study by Collins [24], it was illustrated how the inviting and history-taking of patients’ concerns were shaped by interactions where patients constructed their reply in response to RNs’ invitations. The exploration was linked to several ongoing turns of communication and was formulated and reformulated in the interaction between the RN and the patients, making exploration a dynamic process that was not solely related to what patients expressed. Using checklists and protocols during conversations could both help and hinder the flow of RN–patient communication and the exploration of patient concerns [28, 36]. Such lists were observed to facilitate full coverage of key topics, while at the same time, the interaction could become less natural.

### Observing and assessing patient’s health status

The communication aspects linked to the observing and assessing of patients’ health status includes findings from five of the included studies [23, 26, 28, 33, 35]. These studies suggest that RN communication shifted toward a more collaborative partnership with patients, incorporating both verbal and nonverbal communication strategies to enhance the observation and assessment of patients’ physical state.

During the assessment, the communication style of the RNs seemed to change from starting with an exploration of the patient’s problem into a partnership where observations and assessments were carried out together with the patient and relatives. For example, RNs described what was going to happen by saying “Let’s have a look at you” [33]. Different expressions were used to talk about or explain observations and assessments made and to provide instructions such as “Can you stand up?” [35]. As the consultations continued, RNs needed to provide information about a number of topics, such as weight, blood sugar monitoring, skin care, lifestyle, risk, etc [28].

To understand the patient’s problem and concerns, the RNs were found to both use and observe nonverbal signs and expressions in patient encounters, such as body language and gestures. For example, patient–RN eye contact could decrease across consultations [26]. The RNs also used their nonverbal communication and body gestures to diminish social distance in the interaction with the patient, for example, by getting down on their knees. Such communication was found to be important in mediating an emotional approach to the interaction and ensuring a positive relationship during the examination [23, 33].

### Tailoring the communication

Some studies highlighted the importance of tailoring communication to address patients’ emotional concerns, personal life contexts and individual needs during consultations, including findings charted from eight studies [22–25, 29, 32, 34, 35, 37].

This communication could be used to ensure a more person-centred approach. Showing interest in and being open to patients’ emotional concerns and acknowledging social aspects were described as enabling more individualized interactions [23, 24, 35, 37]. Tailoring the consultation also involved incorporating patient concerns and lifeworld issues into the conversation [23]. Furthermore, RNs adapt their language by avoiding medical terminology and refraining from verbal criticism of the patient, as well as avoiding nonverbal communication that conveys dissent or disapproval [25]. In telephone consultations, this was demonstrated by RNs, who gave the patient time to talk spontaneously [37]. RNs also adapt their communication to local languages to allow for better and more comfortable communication with patients [34].

Patient-centred talk was more dominant than biomedical talk and does not prolong consultation times [22]. The RNs were also found to modify their communication and interaction during consultations, with flexibility towards different patient communication styles. The RNs’ flexibility could resolve any conflicts between patients’ reasons for their visits and their actual needs for treatment [23]. RNs who received video feedback on their consultations were found to increase their focus on the individual patient’s perspective during consultations by paying more attention to the patient’s view and by providing more understandable information [32].

### Facilitating decision-making with patients

The communication skills related to facilitating decision-making processes were mapped in thirteen studies. Facilitating decision-making while acknowledging patient involvement requires RNs to have advanced communication skills to recognize and interpret clinical patterns and understand patients’ needs [22–31, 34–36].

The RNs made a number of decisions during consultations on a daily basis related to assessment, diagnosis, choice and delivery of interventions, and referrals [29]. The RNs commonly seemed to verbalize their reasoning and to check with the patient for confirmation. Decisions were developed through an open dialogue with the patient [27, 28, 35]. In contrast, patient involvement in discussing therapeutic options, planning care, and summarizing the key points of the consultation was infrequent in the study conducted by Almedia et al. [25].

Cooperation and interaction between RNs and patients were important for decision-making and the outcomes of the consultation. Decision-making involved managing patients’ needs, sometimes leading to a mismatch if patients requested something that RNs cannot offer. When co-operation occurred in consultations, decision-making and healthcare advice were easier for the RNs to manage, and patients seemed more satisfied [23]. When the consultation agenda was driven more by RNs than by patients, this could lead to a mismatch in expectations and outcomes. The use of protocols and checklists occasionally posed a risk of neglecting patients’ individual priorities [28]. Cooperation improved when RNs responded to lifeworld issues addressed by patients. The RNs’ reassurance of patients’ expressions and views also facilitated their cooperation [23]. Convergent and divergent behaviours have also been observed in nonverbal communication [26]. For example, laughing, smiling and eye contact were the most common nonverbal signs of joint convergency in RN–patient interactions, and RNs’ computer use was related to divergent nonverbal behaviours.

Decision-making required RNs to have skills in recognizing and interpreting clinical patterns [29, 34], such as the ability and skills to concentrate on complex situations and multiple options simultaneously while gathering the information needed to interpret patients’ signs and symptoms in order to reach a decision [34]. The RNs used different sources of information to underpin their decisions, including their personal experience [29]. More experienced RNs were often found to reach a management decision earlier than their less experienced counterparts [34]. Another study reported that guidelines were rarely used in consultations, while electronic media was sometimes used when the RNs had access to a computer [29].

RN triage and decision-making during telephone consultations were complex when using support systems. The systems seemed to be used to identify which questions should be asked to minimize the risk of missing information and excluding high-urgency conditions rather than supporting the RNs’ decision-making [31]. At times, the RN posed open-ended or issue-specific questions towards the end of the call, offering the patient an opportunity to speak more freely. Additionally, the RNs could adjust the urgency level of the case or consult with a general practitioner if necessary [36]. RNs who used support systems in telephone triage needed to demonstrate sophisticated communication, technological and clinical skills to ensure that patients’ concerns were captured accurately [30].

Decision-making resulted in outcomes that were mainly related to giving health and self-care advice during consultations [23, 27, 28, 35]. In some instances, during telephone triage, the RN summarizes the patient’s medical concerns before inquiring about the specific assistance the caller requires, subsequently providing self-care advice [36]. RNs discussed possible treatment options and provided advice with sensitivity to how much information patients could deal with [27–29, 35] For example, RNs could talk about results and leave patients to show their understanding or ask questions in response [24]. Other techniques included giving back-channel responses to patients’ expressions or using additional verbal and written information [22]. Conversely, in the study conducted by Almedia et al. [25], patients were not given the opportunity to offer feedback to confirm their comprehension.

## Discussion

This review charted study characteristics and findings. The findings were charted and mapped into thematic areas of RN–patient communication. Each of these thematic areas illustrates various communication skills utilized by RNs during primary care consultations. The findings reveal that RNs frequently initiate consultations by actively listening to patients’ descriptions of their health conditions. This approach helped RNs understand patient concerns, build rapport, and facilitate problem solving. Communication skills such as affirmation, summarisation, and diverse questioning techniques were employed to clarify ambiguous responses. In addition, RNs tailored their communication to address patients’ emotional concerns, personal life contexts and individual needs. Similarly, Diamond-Fox and Bone [38], as well as Gustafsson and Wahlberg [39], emphasized that adapting communication to patients’ emotional and social needs is essential for providing person-centred care. Nevertheless, previous research is limited in providing detailed knowledge on the communication skills used to support RNs in clinical practice.

In addition to patient encounters in primary care consultations with RNs, the research summarised in this review focused on communication during telephone triage. In telephone triage, active listening is central, and the use of voice becomes critical for conveying empathy, emotional support, decision-making and confidence, as RNs cannot rely on body language [39]. Decision-support systems used in telephone triage can constrain RNs’ ability to fully utilize their communicative skills. Hence, RNs must be able to balance efficiency with empathy and flexibility, as underscored by Sutton et al. [40].

Currently, there is limited research on the quality and effectiveness of checklists and decision-support systems in this context. Many decision-support systems are designed to provide RNs with predefined options aimed to support their decision-making and triage of patients rather than imposing definitive outcomes [41]. It has been suggested that future advancements in artificial intelligence will offer promising opportunities to support decision-making processes in nursing practice, potentially improving both decision-making processes and overall patient care [42]. Even if technologies can support RNs in their decision-making, they cannot replace RNs. RNs must navigate between providing empathy and making relevant decisions and priorities, as described by Koufidis [43]. The clinical reasoning of RNs when making decisions is multifaceted. It involves intuitively recognizing diagnoses, systematically testing hypotheses, developing innovative solutions to unfamiliar problems, interpreting deviations from expected patterns, and being vigilant in addressing flaw intuition [44]. Additionally, organizational, professional, and linguistic frameworks shape medical practice and influence cognitive habits, which can impact decision-making processes. However, few of the included studies provided a clear and detailed description of RN–patient communication in relation to decision-making. Moreover, research has focused primarily on RNs’ perspectives on decision-making and prioritization of patients’ needs during healthcare visits. Research is needed that explores and recognizes patients’ capabilities, their involvement in decision-making, and the support they require for health literacy.

There are few studies reporting specifically on observed communication and decision-making from nursing practices. When summarised, observed communication research used audio- and video-recordings or participant observations to explore communication behaviours. By systematically observing communication practices, insights can be gained into communication behaviours in different situations and contexts. Such knowledge can support RNs to navigate challenges in practice and underpin their communication in interactions with patients [26]. Research on communication, as observed in clinical practice, provides knowledge with high ecological validity that is needed to inform practices and policy. Additionally, such knowledge could benefit both healthcare providers and their patients. The researchers who performed the current review have learned from previous studies that observed that communication provides different insights than the experiences narrated in interviews. Hence, this indicates a need to expand the area of research on communication using different forms of observations, including audio and video recordings of communication in RNs’ encounters with patients in clinical practice.

The current review indicates that there are few observational studies on communication and decision-making in primary care RN consultations, which is different from research on physician–patient communication [45]. Knowledge of physician–patient communication has been developed into evidence-based structures for teaching and analysis of such communication skills, which are widely used in medical schools [46]. This is in contrast to nursing education, where more education and training within this area are needed [47]. Current communication models rely mainly on the physician’s role and responsibility, such as the Calgary–Cambridge Guide and its enhanced versions [5, 48]. However, RN communication, as summarised in this review, seems to follow a similar structure, even though this was not explicitly stated in the studies. Communication skills are considered fundamental in RN consultations [38], and questions remain in terms of whether current frameworks could be used as guides for RN–patient consultations in primary care, even if they were developed for medical schools.

In the studies being reviewed, RNs were reported to adapt their communication according to patients’ communication style and the manner in which the patients articulated their problems. However, this ability is not consistently evident in the results. RNs’ communication skills are integral to person-centred care, which underscores the importance of RNs’ involvement in patient care to strengthen relationships where all parties are engaged [38, 49, 50]. Communication skills are imperative for understanding patient needs and delivering person-centred care. The attributes of person-centred communication involve communication skills related to recognizing, inviting and involving the patient, requiring RNs to be attentive and responsive in interactions [51]. The findings indicate that RNs integrate these aspects, resulting in efficient care, as described by McCance and McCormack [52]. However, there is limited empirically based research, and the current research reviewed did not offer any structured communication models for guidance in RNs’ assessments and decision-making.

## Strengths and limitations

This review has several strengths. First, the review protocol was registered, enhancing the methodological rigour. Second, multiple databases were searched for relevant studies in accordance with the aim. The rigorous process undertaken to perform a thorough search included help from a specialist librarian and the use of a digital web tool, Rayyan. Another strength of the study was the collaboration of several authors, who screened and assessed the papers together to reach a consensus. Additionally, PRISMA flow chart, were used to ensure transparency and methodological rigor, by enabling a step-by-step account of the study selection process to be followed, which improved the credibility of the results. To ensure rigor and quality for this scoping review, the different stages of the processes have been described as thoroughly as possible [15]. However, this scoping review has some limitations. Despite the use of a comprehensive search strategy, there is always a risk that some relevant studies will have been missed. Second, the inclusion of only English-language studies may have resulted in missing relevant articles published in other languages. There were also studies using multiple data source in which the findings could not be clearly derived from observational data alone. Finally, few studies could be included in the results because of the variation in results and the fragmented picture of the RNs’ interactions and decision-making in primary care. This finding is consistent with that of Norful et al. [18], who reported that there is still great variability in nursing roles and responsibilities in primary care, which points to the importance of further research in this area.

## Conclusions

This overview of RN–patient communication and decision-making in primary care revealed that RNs utilized various communication skills during different phases of consultations. A lack of comprehensive studies on this topic has been identified. Few studies have described communication in these patient encounters in detail. This scarcity highlights the need for further in-depth and comprehensive studies on RN–patient communication in primary care to inform both clinical practices and nursing education. While this review provides some guidance, more research is necessary to inform policy and practices.

## Supplementary Information

Below is the link to the electronic supplementary material.

Supplementary Material 1

## Data Availability

All the data generated or analysed during this study are included in this published article.

## Abbreviations

PAGER: Patterns, advances, gaps, evidence for practice and research recommendations
PRISMA: Preferred reporting items for systematic review and meta-analysis
PRISMA-ScR: Preferred Reporting Items for Systematic Reviews and Meta-analyses Extension for Scoping Reviews
RN: Registered nurses
