# Supplementary material for: Registered nurse–patient communication and decision-making in primary care consultations: a scoping review
Source: BMC Nurs. 2026 Jun 23;25:562. doi: 10.1186/s12912-026-04937-w (PMC13295847; doi:10.1186/s12912-026-04937-w)
Supplement: Supplementary file 1 — Supplementary Material 1 [file 12912_2026_4937_MOESM1_ESM.docx]

Search string Decision making, Clinical reasoning, Nurses, Primary care and healthcare communication

Cinahl *n*=2842

PubMed *n*=4345

Scopus *n*=2525

Web of science *n*=2534

Total 12066

After removal of duplicates 7980 records were added to Rayyan.

| Database: Cinahl via Ebsco Host, 20 mars 2025 | | |
| --- | --- | --- |
| 1 | (MH "Decision Making+") OR (MH "Decision Making, Clinical+") OR (MH "Clinical Reasoning") OR (MH "Nursing Assessment") | 163,807 |
| 2 | (TI "decision making" OR TI "clinical reasoning" OR TI "health communication" OR TI "nursing assessment" OR TI consultation* ) OR (AB "decision making" OR AB "clinical reasoning" OR AB "health communication" OR AB "nursing assessment" OR AB consultation* ) | 123,880 |
| 3 | 1 OR 2 | 247,803 |
| 4 | (MH "Nurses+") OR (MH "Nurse-Patient Relations") OR (MH "Nursing Role") | 287, 368 |
| 5 | AB nurse* OR TI nurse* | 377 767 |
| 6 | 4 OR 5 | 516,496 |
| 7 | (MH "Primary Health Care") OR (MH "Family Practice") OR (MH "Community Health Nursing") | 118 339 |
| 8 | (TI "primary health care" OR TI "primary healthcare" OR TI "primary care" OR TI "primary health cent*" ) OR ( AB "primary health care" OR AB "primary healthcare" OR AB "primary care" OR AB "primary health cent*" ) | 98 125 |
| 9 | 7 OR 8 | 166 792 |
| 10 | 3 AND 6 AND 9 | 3251 |
|  | Filter years 1998-2025, English language, Peer reviewed | 2842 |

## **Formatted search string:**

((MH "Decision Making+") OR (MH "Decision Making, Clinical+") OR (MH "Clinical Reasoning") OR (MH "Nursing Assessment") OR TI "decision making" OR TI "clinical reasoning" OR TI "health communication" OR TI "nursing assessment" OR TI consultation* OR AB "decision making" OR AB "clinical reasoning" OR AB "health communication" OR AB "nursing assessment" OR AB consultation*)) AND ((MH "Nurses+") OR (MH "Nurse-Patient Relations") OR (MH "Nursing Role") OR AB nurse* OR TI nurse* ) AND (( AB "primary health care" OR AB "primary healthcare" OR AB "primary care" OR AB "primary health cent*" ) OR ( TI "primary health care" OR TI "primary healthcare" OR TI "primary care" OR TI "primary health cent*" ) OR ( (MH "Primary Health Care") OR (MH "Family Practice") OR (MH "Community Health Nursing"))

| Database: Pubmed, 20 mars 205 | | |
| --- | --- | --- |
| 1 | "Decision Making"[Mesh] OR "Clinical Decision-Making"[Mesh] OR "Clinical Reasoning"[Mesh] OR "Health Communication"[Mesh] OR "Nursing Assessment"[Mesh] | 280 390 |
| 2 | "Decision Making"[tiab] OR "Clinical Reasoning"[tiab] OR "Health Communication"[tiab] OR “Nursing assessment”[tiab] OR Consultation*[tiab] | 295 221 |
| 3 | 1 OR 2 | 515 877 |
| 4 | "Nurses"[Mesh] OR "Nurse-Patient Relations"[Mesh] OR "Nurse's Role"[Mesh] | 159 227 |
| 5 | Nurse*[tiab] | 320 571 |
| 6 | 4 OR 5 | 398 292 |
| 7 | "Primary Health Care"[Mesh] OR "General Practice"[Mesh] OR "Primary Care Nursing"[Mesh] | 260 067 |
| 8 | “primary health care”[tiab] OR “primary healthcare”[tiab] OR “primary care”[tiab] OR “primary health cent*”[tiab] | 174 919 |
| 9 | 7 OR 8 | 354 635 |
| 10 | 3 AND 6 AND 9 | 4791 |
|  | Filter years 1998-2025  English language | 4345 |
|  |  |  |

## **Formatted search string:**

**((("Nurses"[Mesh] OR "Nurse-Patient Relations"[Mesh] OR "Nurse's Role"[Mesh]) OR (Nurse*[tiab])) AND (("Primary Health Care"[Mesh] OR "General Practice"[Mesh] OR "Primary Care Nursing"[Mesh]) OR ("primary health care"[tiab] OR "primary healthcare"[tiab] OR "primary care"[tiab] OR "primary health cent*"[tiab]))) AND (("Decision Making"[Mesh] OR "Clinical Decision-Making"[Mesh] OR "Clinical Reasoning"[Mesh] OR "Health Communication"[Mesh] OR "Nursing Assessment"[Mesh]) OR ("Decision Making"[tiab] OR "Clinical Reasoning"[tiab] OR "Health Communication"[tiab] OR "Nursing assessment"[tiab] OR Consultation*[tiab]))**

| Database search 20 mars 2025 | | |
| --- | --- | --- |
| Pubmed | 1998- 20 mars 2025 | *4345* |
| Cinahl | 1998- 20 mars 2025 | *2842* |
| Web of science | 1998- 20 mars 2025 | *2354* |
| Scopus | 1998- 20 mars 2025 | *2525* |
| Summa dessa fyra sökningar: |  | 12066 |
| Deleted doublets in endnote: | 4086 remowed | 7980 reamining – to Rayyan |

## **Formatted search string:**

**(("2023/05/01"[Date - Entry] : "3000"[Date - Entry])) AND (((("Nurses"[Mesh] OR "Nurse-Patient Relations"[Mesh] OR "Nurse's Role"[Mesh]) OR (Nurse*[tiab])) AND (("Primary Health Care"[Mesh] OR "General Practice"[Mesh] OR "Primary Care Nursing"[Mesh]) OR ("primary health care"[tiab] OR "primary healthcare"[tiab] OR "primary care"[tiab] OR "primary health cent*"[tiab]))) AND (("Decision Making"[Mesh] OR "Clinical Decision-Making"[Mesh] OR "Clinical Reasoning"[Mesh] OR "Health Communication"[Mesh] OR "Nursing Assessment"[Mesh]) OR ("Decision Making"[tiab] OR "Clinical Reasoning"[tiab] OR "Health Communication"[tiab] OR "Nursing assessment"[tiab] OR Consultation*[tiab])))**
